# Partitioned gradient-index phononic crystals for full phase control

**DOI:** 10.1038/s41598-020-71397-w

**Published:** 2020-09-03

**Authors:** Jaeyub Hyun, Miso Kim, Wonjae Choi

**Affiliations:** 1 AI Metamaterial Research Team, Korea Research Institute of Standards and Science (KRISS), 267 Gajeong-ro, Yuseong-gu, Daejeon, 34113 Republic of Korea; 2 Present Address: Structural Engineering Department, Jacobs School, University of California San Diego (UCSD), 9500 Gilman Dr, La Jolla, CA 92093 USA

**Keywords:** Engineering, Mechanical engineering

## Abstract

Gradient-index phononic crystals (GRIN-PC), characterized by layers with spatially changing refractive indices, have recently been investigated as part of the effort to realize flat lenses in acoustic and elastic regimes. Such gradient-index lens must be inversely designed from the corresponding refractive indices in order to manipulate the target wave. Unfortunately, estimating the index of this type of lens is not straightforward and requires substantial iterative computation in general, which greatly limits the applicability of GRIN-PC to flat lenses. In this work, we propose a novel design of a GRIN-PC in which neighboring layers are separated by partitions, thus preventing waves in each layer from interacting with other layers. This partitioned GRIN-PC design enables us readily to control the phase gradient accurately at the lens’ end, resulting in direct calculation of indices for target wave manipulation. A detailed methodology for partitioned GRIN-PC based collimator and Bessel-beam generator is proposed and experimentally validated to confirm the versatile use of our design in wave engineering applications.

## Introduction

Phononic crystals are artificially designed periodic structures whose effective mechanical properties are designed to have unprecedented wave phenomena^1^. For this reason, they have established novel routes of wave modulation in physics and engineering fields. Phononic crystals have been investigated in relation to a number of applications, such as wave focusing^2–6^, negative refractive index lens^7–9^ and bandgap material^10–14^. Other recent work has attempted to combine phononic crystals with energy harvesting^5,15,16^.

The gradient-index phononic crystal (GRIN-PC) lens is a type of phononic crystal system consisting of a number of layers with different refractive indices, making it capable of modulating acoustic wave paths. The GRIN lens concept has been known for many years in optics^17–20^, as it offers the advantage of enabling the creation of a flat lens in places where the use of a typical curved lens is limited. This system has also been explored for use in acoustic applications^3,21,22^. Focusing wave energy into a focal area is one example of the use of a flat GRIN lens. Gradually changing indices in a focusing lens can readily be designed via the hyperbolic secant profile function^23^. However, most of the methods in the existing studies on GRIN-PC systems thus far have been confined to focusing the wave energy using the hyperbolic-secant index profile^2,4,22,24–28^, because of its simplicity, thus restricting design of GRIN-PC with any other wave path profile for general purposes.

Finding the indices of a GRIN lens for general purposes is not always as straightforward, compared to the use of a hyperbolic-secant focusing lens. The hyperbolic-secant function is an analytical solution for the indices of the focusing lens, which is, admittedly, an exceptional case rather than a general one^23^. In general, the index of a layer in a GRIN lens does not have a one-to-one relationship with the phase shift in the same layer because the wave incident to a layer undergoes a change of direction over the neighboring layers. Thus, analytically calculating the indices for target phases is difficult without approximations. Moreover, calculating the indices commonly requires tedious and substantial iterative numerical computations^23^. If the index in the GRIN-PC system is readily and accurately designed to control the target phase at the lens’ end without the need for iterative calculations, this advance will open a new avenue not only for wave-focusing but also for the general wave-modulation applications using the GRIN-PC lens.

In this paper, we introduce a novel GRIN-PC design that utilizes the concept of a partition, which enables facile estimations of the refractive indices for wave manipulation without the need for complex iterative computations. Furthermore, a methodology is proposed to implement the design for two practical wave-guiding applications: A collimator and a Bessel-beam generator using the new partitioned GRIN-PC concept are successfully designed and experimentally demonstrated to prove the applicability of the proposed design and methodology for general-purpose GRIN-PC systems.

## Results

### Partitioned gradient-index phononic crystals

In order to link and control the phase at the transmitted end of the lens directly using the refractive indices in the gradient-index phononic crystal (GRIN-PC), we introduce partitions between the neighboring layers in the GRIN-PC system. By having extremely different impedance property from the medium (e.g., air in slits vs. aluminum plate), the “partitions” can prohibit the propagating waves in each layer from interfering with those in the neighboring layers. Note that a partition is often used for metasurfaces^29^ to divide neighboring sections as well, but this concept has not been used for a GRIN-PC system to the best of the authors’ knowledge. The partitioned GRIN-PC (pGRIN-PC) design allows the generalized Snell’s law, commonly used when designing metasurfaces^2,4,22,24–28^, for PC-designers to readily calculate the phase shifts based on the target wave modulation. Hereafter, we will use the term “phase shift” for the phase difference between the incident- and the transmitted- sides of the lens and “phase difference” for the others such as difference between phases at the transmitted (right-hand-side) end of a layer and at the same end of the center layer (or the 1st layer) of a flat lens.

**Figure 1:**
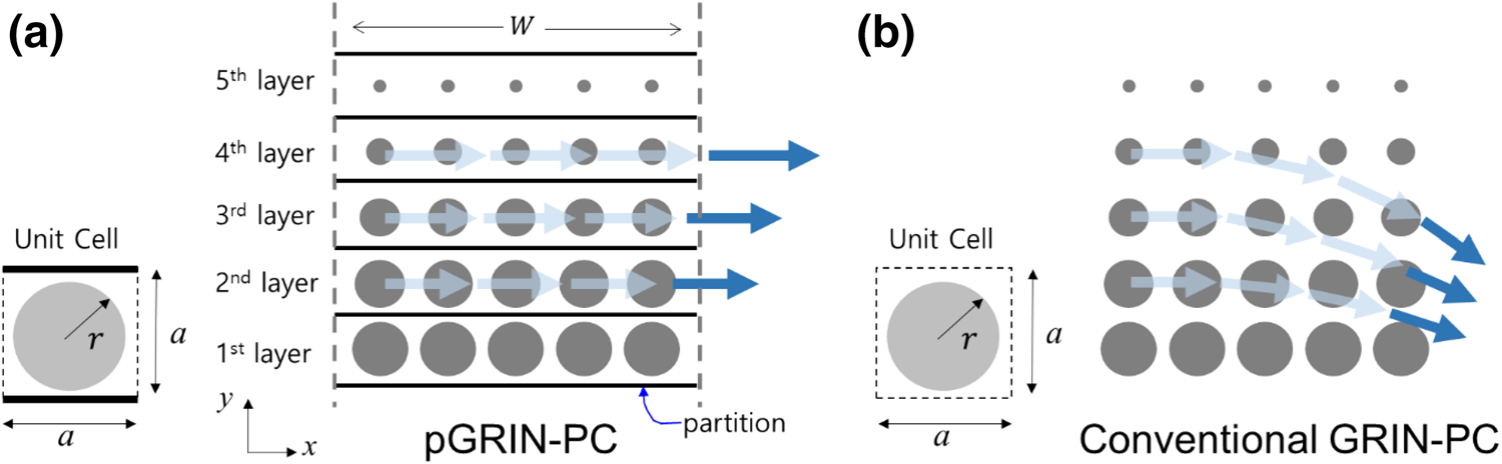
Gradient-index phononic crystals and associated unit cells: (**a**) Proposed design with partitions, and (**b**) the conventional design. Wave paths in the PCs are indicated by arrows. Unit cells are shown for both cases with a circular inclusion with radius $r$.

Figure 1a,b show the pGRIN-PC system and its conventional counterpart without a partition, respectively, with each layer containing five unit cells in both systems. In pGRIN-PC, the square unit cell consists of a hole with radius *r* in the center and partitions on the upper and lower sides on an aluminum plate with a thickness of 2 mm, as shown in Fig. 1a. These partitions guide the incident wave, causing it to propagate only in the *x* direction, while Fig. 1b shows the wave paths are bent due to the index difference in the neighboring layers of the conventional GRIN-PC. Hence, the phase shift ${\phi }_{\mathrm{PC}}$ in a layer between the incident and the transmitted sides (as indicated by the arrows in Fig. 1a) can be readily calculated via

1 $$ \phi_{{{\text{PC}}}} = n_{{{\text{PC}}}} k_{0} W, $$

where ${n}_{\mathrm{PC}}$ is the effective refractive index, ${k}_{0}$ is the wavenumber of the surrounding medium, and *W* is the width of the pGRIN-PC. If we want a particular phase at the transmitted end of the lens, the corresponding indices can readily be computed by Eq. (1) for the pGRIN-PC case.

### Systematic design process of pGRIN-PC systems

The entire design process to model the pGRIN-PC consists of three steps: (1) setting the refractive index range, (2) calculating the width to cover a full phase from 0 to 2π, and (3) finding the inclusion radii in the layers for target purposes depending on the design, here, a collimator. It would be ideal for the partition to have a width of zero; however, due to manufacturability issues, a through-cut with a 0.5 mm width is chosen instead, and thus the through-cut line of 0.5 mm widths are utilized as the partitions. Accordingly, the sizes of the upper and lower partitions in one unit cell are both 0.25 mm. The target frequency is 50 kHz, and the corresponding wavelength $\uplambda$ is approximately 18.7 mm for flexural wave (A0 Lamb wave). The size of the unit cell, *a*, is set to 5 mm ($\sim\uplambda /4)$.

**Figure 2:**
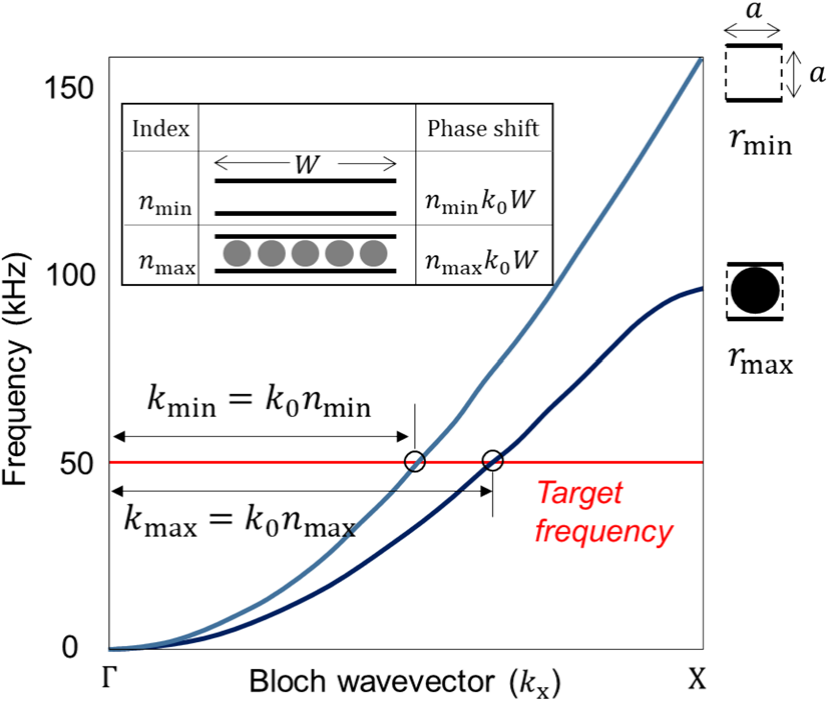
Band structures for the minimum and maximum radii to find their effective indices, as calculated by simulations for the design of the unit cell for the collimator. Phase shifts for the two sample layers are indicated in the inset.

First, in order to find the index range, the minimum and maximum indices are computed. Because the radius *r* is the only parameter to be controlled in the index design, the minimum and the maximum radii of the unit cell define the index range applied to the pGRIN-PC lens. It is reasonable to set the minimum radius to zero, whereas the maximum value is set to the largest achievable radius of 1.9 mm, considering manufacturability, and the corresponding unit cells are shown in Fig. 2. Note that these values can differ depending on the PC and the environment. Once the two radii are selected, the corresponding effective wavenumbers can be, for example, obtained from the band structures computed by COMSOL multiphysics, as shown in Fig. 2. Then, their effective refractive indices at the target frequency can be calculated using the equation

2 $$ n_{{{\text{PC}}}} = k_{{{\text{PC}}}} /k_{0} , $$

where ${k}_{\mathrm{PC}}$ is the effective wavenumber of the unit cell. At 50 kHz, the wavenumbers obtained for the radii 0 and 1.9 mm in Fig. 2 are 327.24 and 400.57 [1/m], respectively, and their refractive indices are 1 and 1.2237, which defines the limits for the index range.

Second, it is necessary to calculate the width *W* to cover a full phase from 0 to 2π. Note that, in the pGRIN-PC case, the phase difference $\Delta {\phi }_{\mathrm{min}}^{\mathrm{max}}$ resulting from unit cells with the two extreme radii are usually smaller than 2 $\uppi$,$\Delta {\phi }_{\mathrm{min}}^{\mathrm{max}}=({n}_{\mathrm{max}}-{n}_{\mathrm{min}}){k}_{0}a<2\pi$, because an index change in one unit cell of a phononic crystal is much smaller than, for example, that in resonance-based metamaterials. Therefore, one layer should consist of the number of unit cells that results in a 2π difference, and the width *W* of the layers becomes integer multiples of the unit cell size.

The width *W* of all layers in the pGRIN-PC system must be identical to the lens width. When waves propagate to a distance *W*, the difference of the phase shifts in the layers of the minimum and maximum refractive indices can be calculated as $({k}_{\mathrm{max}}-{k}_{\mathrm{min}})W$, which should be 2 $\uppi$ to cover the full phase. By comparing the difference and the refractive index in Eq. (2), the width *W* can be expressed as:

3 $$ W = \frac{\lambda }{{n_{{\max}} - n_{{\min}} }}, $$

where $\lambda =2\uppi /{\mathrm{k}}_{0}$ is the wavelength in the surrounding medium. The width obtained from Eq. (3) is 83.5 mm for the collimator, and the layer is selected to have 17 unit cells; thus, the layer width is expressed as *W* = 85 mm (= 17 unit cells × 5 mm/unit cell), as close as possible to 83.5 mm.

The third step is to find the indices for the layers other than the two layers with the minimum and maximum radii and then to determine their corresponding geometrical parameters, i.e., the hole radius *r*. Note that the three-step design methodology is utilized mainly for designing phononic crystals, although larger in size, which operates in a wider frequency range than the metamaterials^30^. In the pGRIN-PC case, the indices are readily found when the target wave directions are set, whereas designing such a conventional GRIN-PC system with the same profile requires tremendous computational labor to find them. Once the indices are selected, finding the corresponding radii is an inverse design process. In order to find the shape and size of the inclusion for the target refractive index, a number of methods can be used, such as trial/error^31^, optimization^29,32–34^ and machine learning^35^. In this paper, we derive a fifth-order polynomial to link the refractive indices to the radii and use it to find the hole radii. The unit cell of a GRIN-PC lens often has a simple inclusion geometry, as shown in Fig. 1, and a polynomial for such a shape can readily be found with fewer sample points than the number required for the trial/error method^31^.

In order to derive the equation, ten different radius values between 0.3 to 1.9 mm were selected, and their refractive indices were computed by Eq. (2) with the effective wavenumbers from the band structures, as indicated in Fig. 2. From the ten radius-index relationships, we derive a fifth-order polynomial by means of the least square method:

4 $$ 10^{ - 4} r = 1.35n^{5} - 7.79n^{4} + 17.95n^{3} - 20.66n^{2} + 11.89n - 2.74 $$

Note that Eq. (4) can be used for designing not only for the collimator example but also for a pGRIN-PC system for any wave modulation, such as the Bessel beam or focusing types. Provided the refractive indices, we are then able to find the radius sizes with the equation.

### Applications of pGRIN-PC and experimental realization

**Figure 3:**
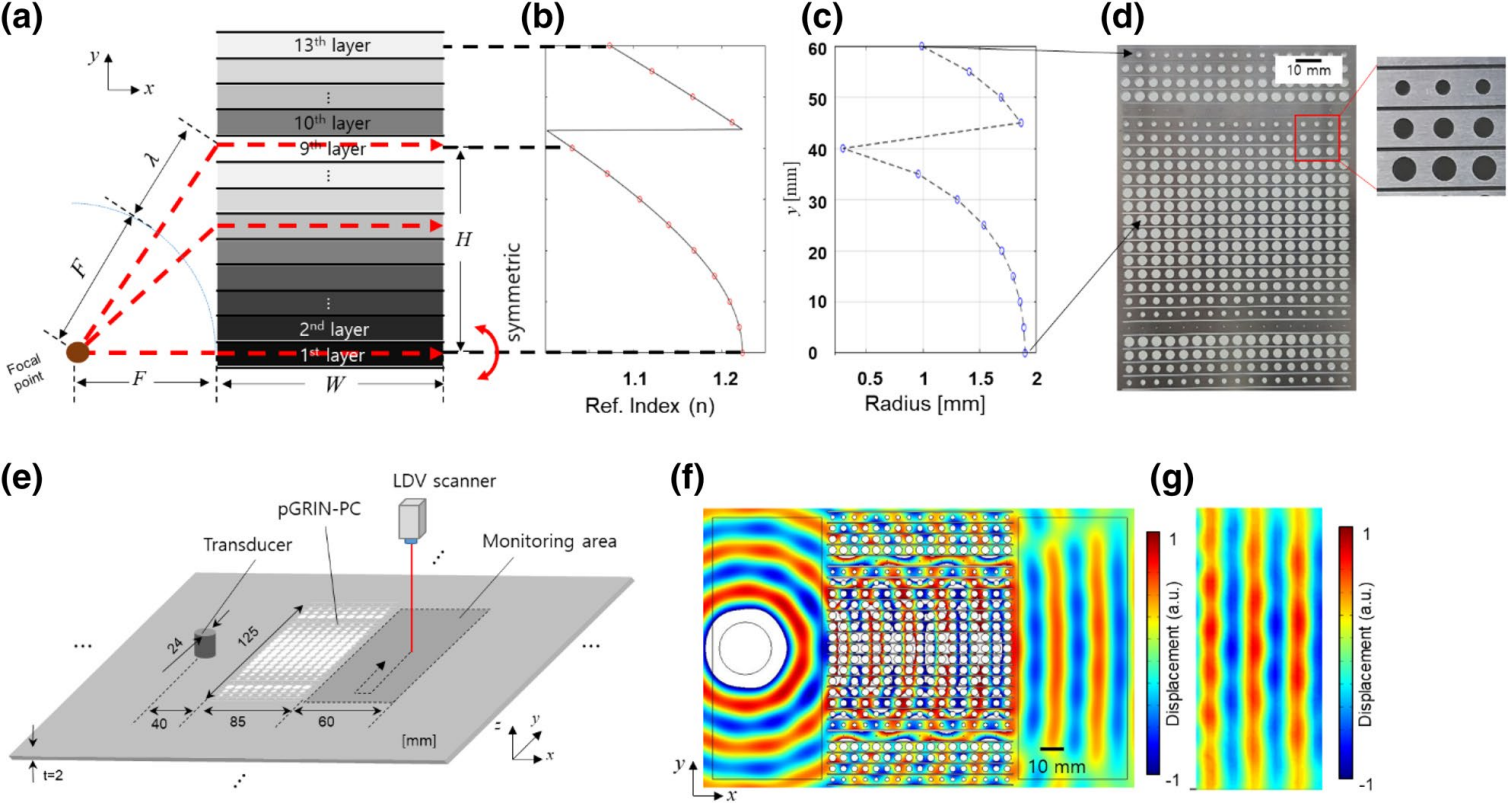
Simulated and experimental results for the collimator design: (**a**) geometry used when calculating the indices of each layer, (**b**) refractive indices calculated for the layers (**c**) corresponding radii, (**d**) manufactured collimator using the pGRIN-PC system, (**e**) experimental setup, (**f**) simulation result, and (**g**) experiment results of the pGRIN-PC collimator. The wave-fronts in the monitoring area are in-phase in both the simulation and the experiment.

As a representative example, a pGRIN-PC based collimator is designed to create a plane wave from a cylindrical source. For the collimator design, phase shift from the source point to the right-side of all layers in the pGRIN-PC must be equal as the red dashed-lines in Fig. 3a, from which the refractive indices of the layers can be calculated as in Fig. 3b. The corresponding radii for the layers in the pGRIN-PC system is computed by Eq. (4) as indicated in Fig. 3c (detailed equations are presented in the supplementary notes). Note that the refractive index in Fig. 3b shows an abrupt jump between the 9th and 10th layers, since the target phase required for the layers above the 10th layer are larger than 2 $\uppi$, in which case we reset the target phase to be remainder of the original one divided by $2\uppi$.

The designed pGRIN-PC for the collimator is then manufactured in Fig. 3d and experimentally characterized for validation. The experimental setup is shown in Fig. 3e. A transducer with a 50 kHz resonance frequency is attached to the plate, and a laser Doppler vibrometer (LDV) scans the flexural displacement normal to the plate. Note that a rather large $1.8 m\times 1.2 m$ aluminum plate is used for the pGRIN-PC being manufactured on in order to separate the unwanted waves reflected from the plate boundaries and the waves of interest transmitted from the pGRIN-PC. Detailed descriptions of this setup are provided in the supplementary note. In parallel with the experiments, harmonic simulations are conducted using COMSOL Multiphysics. In Fig. 3f,g, the simulation and the experimental results are in good agreement, showing that the outgoing waves are in-phase. The large radius difference between the 9th and the 10th layers as in Fig. 3c seems to lessen the lens’ performance due to the impedance mismatch, which can be witnessed in Fig. 3f, but enhancing the lens’ quality will be left for the future work. Nevertheless, the plane wave is clearly shown to propagate in Fig. 3f,g, demonstrating that the wave is successfully collimated. In order to ensure the full phase controllability of the proposed pGRIN-PC for the collimator, we performed additional experiment where focusing is observed upon the incident waves on the collimators Fig. S7, which is fully described in the Supplementary Note.

In addition, in order to illustrate the further applicability of the pGRIN-PC system, we applied our pGRIN-PC design methodology to another example, in this case, a Bessel beam generator. A Bessel beam is a beam with a high aspect-ratio beam size. It can be created in theory by two plane waves directing opposite angles $\beta$ and $-\beta$, implying that the wave along the mirror axis does not spread out.

The unit cell configuration for this example is selected to be identical to that used for the collimator design, as shown in Fig. 1a. Thus, if the target refractive index for the Bessel beam generator is chosen, its target radii are readily found using Eq. (4). The Bessel beam in this paper is designed to have a refractive angle of $\beta ={20}^{\circ }$, and the indices of the layers increase linearly from the center layer in order to create two plane waves directing opposite angles ${\pm 20}^{\circ }$. In this Bessel beam case, we set the maximum radius to 1.66 mm, different from the collimator case, in order to show that the pGRIN-PC system can be built with other index ranges when using the proposed design methodology. In this case, the corresponding index is 1.16, the width is then calculated and found to be 116.8 mm, and thus *W* is set to have 24 unit cells such that 24 ea. × 5 mm = 120 mm.

**Figure 4:**
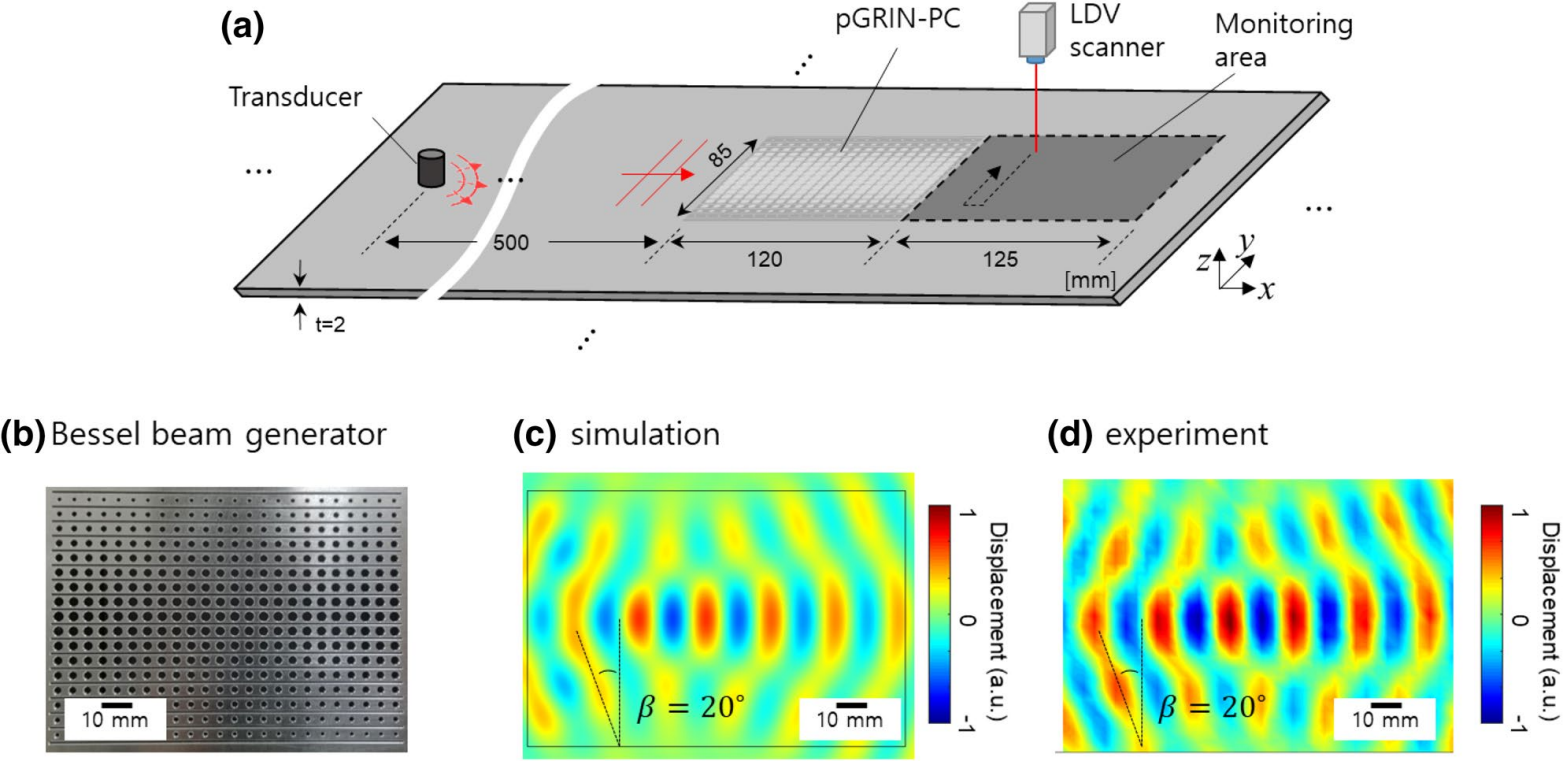
Bessel-beam generator created with the pGRIN-PC: (**a**) experiment setup, (**b**) manufactured specimen, and (**c**) simulated and (**d**) experimental results.

Figure 4a presents the experimental setup for the Bessel-beam generator, and Fig. 4b shows the fabricated Bessel beam generator based on the pGRIN-PC design. The simulation result in Fig. 4c clearly visualizes the function of the Bessel beam generator with the targeted ${20}^{\circ }$ angle. Figure 4d experimentally confirms the performance of the Bessel beam generator, which is also in good agreement with the simulated results in Fig. 4c given the ${20}^{\circ }$ refraction angles.

## Discussion

In conclusion, we proposed a design for a gradient index phononic crystal with a partition (pGRIN-PC) in order to achieve full phase control and thus readily to design a general-purpose GRIN-PC system for wave modulation. With the pGRIN-PC design, in contrast to the conventional GRIN-PC case, we can calculate the target refractive index of each layer without a complicated calculation process and can thus efficiently realize phononic crystals for wave modulation. Notably, introducing partitions into PC unit cells makes it possible to achieve the following two approaches; (1) The optimal number of unit cells required to cover the full phase range of $2\pi$ can be determined. (2) It is possible to associate the GRIN concept with the generalized Snell’s law commonly used in metasurfaces. In addition, a facile design methodology for realizing the pGRIN-PC system is suggested and applied to two representative practical applications: a collimator and a Bessel beam generator in an aluminum plate. These two examples are numerically and experimentally demonstrated, proving that the proposed phononic crystal can modulate the wave path to any target direction and that the pGRIN-PC design has the potential to be implemented in limitless applications. Accordingly, the proposed pGRIN-PC design enables the applicability of a scattering-based gradient index phononic crystal to a fully controllable flat lens.

## Methods

### Time-harmonic finite element simulation of pGRIN-PC systems

The finite element commercial software package, COMSOL Multiphysics, is utilized for the time-harmonic analysis of the constructed pGRIN-PC systems. More specifically, we choose the three-dimensional (3D) solid stress–strain application mode in structural mechanics module in COMSOL. The allowable maximum mesh size is selected as the 1/10 of the wavelength ($\lambda /10$) in order to recover enough the spatial resolution of wave propagation. The material used in the simulations is aluminum: The mass density, the Young's modulus, and the Poisson's ratio are set to $\rho =2631.4 \mathrm{kg}/{\mathrm{m}}^{3}, E=70 \mathrm{GPa},$ and $\nu =0.33$, respectively, which are the nominal values at 1 atm and 20 °C. The input source transducer is approximated by a unit force in the *z*-direction to excite only the flexural displacement. A time-harmonic analysis is conducted at 50 kHz operational frequency to compute the flexural wave fields generated through the constructed pGRIN-PC systems, such as the collimator and the Bessel beam generator. Perfectly matched layers (PMLs) are set on the exterior boundaries of the simulation domain to eliminate boundary-reflected elastic waves.

### Experimental setup

Aluminum plate with the thickness of 2 mm is used, and it has the dimension of $1.8\mathrm{ m }\times 1.2 \mathrm{m}$. The pGRIN-PC systems constructed here, such as collimator and Bessel beam generator, are fabricated on the center area of the aluminum plate. Experimental setup is composed of two parts for wave excitation and wave visualization. The wave excitation part consists of a function generator (AFG3051C, Tektronix), a power amplifier (7,224, AE Techron), and piezoelectric transducer customized to have a resonant frequency of 50 kHz (Ceracomp Co. Ltd.). Then, for the wave visualization part, the LDV (PSV-400, OFV-5000, Polytec) is used to measure the time-dependent displacement fields. A tone burst signal with 15 cycles with the pulse-duration of 100 ms at 50 kHz. The amplitude of the input signals is set to peak-to-peak voltage of 10 V. 30 averages of the signals are carried out in order to guarantee enough high signal-to-noise ratio (SNR).

See the supplementary material for (S1) the process to design a collimator and (S2) a Bessel beam generator, and (S3) the detailed experimental setup. See also the supplementary material Video 1 and 2 for the experimental results.

## Supplementary information

Supplementary information 1

Supplementary information 2

Supplementary information 3
