# Supplementary material for: Partitioned gradient-index phononic crystals for full phase control
Source: Sci Rep. 2020 Sep 3;10:14630. doi: 10.1038/s41598-020-71397-w (PMC7471306; doi:10.1038/s41598-020-71397-w)
Supplement: Supplementary file 1 — Supplementary information 1 [file 41598_2020_71397_MOESM1_ESM.docx]

**Supplementary Note for**

**Partitioned gradient-index phononic crystals**

**For full phase control**

**Jaeyub Hyun^1,2^, Miso Kim^1*^, and Wonjae Choi^1*^**

^1^Center for Safety Measurement, Korea Research Institute of Standards and Science (KRISS),

267 Gajeong-ro, Yuseong-gu, Daejeon 34113, Republic of Korea

^2^Present address: Structural Engineering Department, Jacobs School, University of California San Diego (UCSD), 9500 Gilman Dr., La Jolla, CA 92093, USA.

^*^Corresponding author, E-mail Address: misokim@kriss.re.kr, w.choi@kriss.re.kr

In Supplementary Note S1, a detailed process to design a collimator is described with pGRIN-PC. A Bessel beam generator is also demonstrated in the manuscript and its design process is described in S2. In S3, the experimental setup is presented for measuring wave behavior with the collimator. In addition, animations of the experimental results with the collimator and the Bessel beam can be found in the Supplementary Material 1 and 2.

**S1. Designing a collimator**

The refractive indices of the layers in a collimator can be calculated by the simple geometrical descriptions in Fig. S1, and Eq. (S1a) is directly obtained from the figure. For the collimator design, phase differences from the source point to the right-side of all layers in the pGRIN-PC must be equal, which is described in Eq. (S1b). From Eq. (S1a), the height covering 2$\pi$ is calculated as $H=\sqrt{\lambda^{2}+2\lambda F}$, and the refractive index can be found by comparing Eq. (2), (S1a) and (S1b). The refractive index $n(y)$ is described in Eq. (S1c) as a function of the location *y*. Furthermore, the discrete form of the refractive index $n_{m}$ can also be obtained as Eq. (S1d) since the collimator is actually an array of the unit cells as shown in Fig. S1, and Fig S2 shows the refractive indices computed for the layers of the collimator. As the size of the layer in the *y*-direction is equal to the size of the unit cell *a*, *a* is used in equation (S1d). The fifth-order polynomial in Eq. (4) is shown in Fig. S2, and the corresponding radiuses are computed for the layers in Fig. S2.

$H^{2}+F^{2}=\left( F+\lambda\right)^{2}$ and $y^{2}+F^{2}=\left( F+s \right)^{2}$ (S1a)

$k_{0}\left( F+s \right)+\phi\left( y \right)=k_{0}F+\phi(0)$ (S1b)

$n\left( y \right)=\frac{\sqrt{y^{2}+F^{2}}-F}{W}+n(0)$ (S1c)

$n_{m}=\frac{\sqrt{a^{2}\left( M-m \right)^{2}+F^{2}}-F}{W}+n_{M}$ (S1d)

**
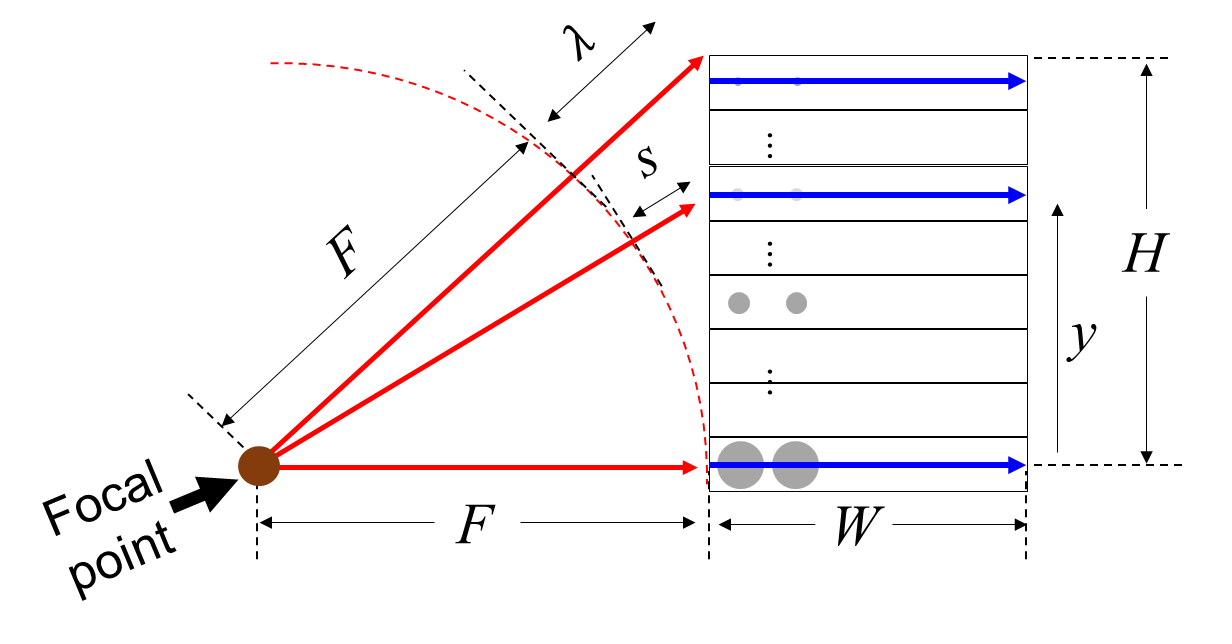
**

**Figure S1.** Geometrical descriptions for the refractive index computation in the collimator design using pGRIN-PC.

In order to verify the target phase change in each layer, time-harmonic simulations at 50 kHz using COMSOL Multiphysics are conducted for each layer consisting of the geometrical parameters (radiuses) calculated. In this simulation, each layer has 17 unit-cells with one radius size. The collimator is designed to create a plane wave from a cylindrical source. Using reciprocal theory [1], the performance can be validated with a plane wave input by whether the wave is propagated toward the source location. Fig. S3 shows the result with a plane wave input from the left-hand side, and the outgoing wave shows to agree well with the target indices.

**
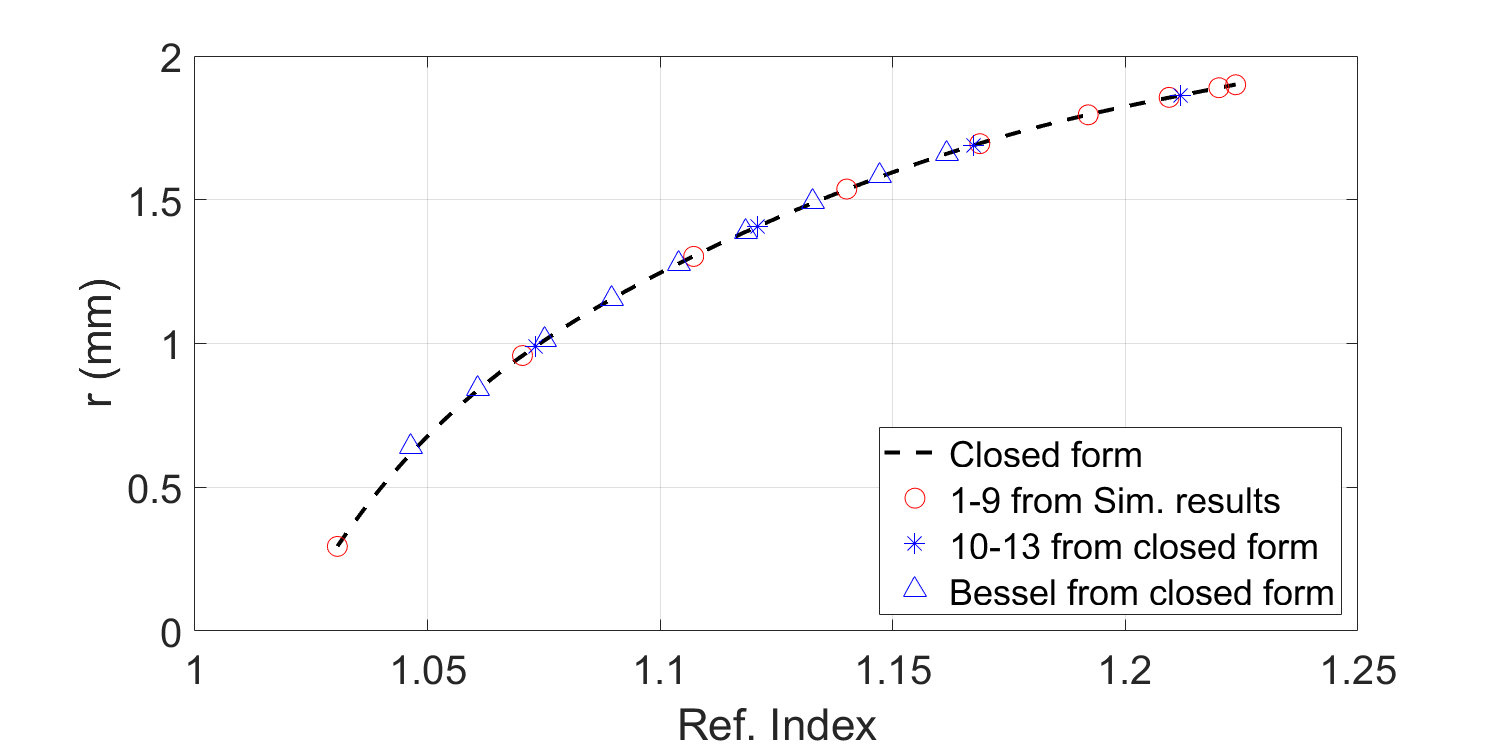
**

**Figure S2.** Radius design using the closed form. The close form (black dashed line), radiuses from the 1^st^ to the 9^th^ layers (red circles) and from the 10^th^ to the 13^th^ layers (blue stars) for the collimator, and radiuses for the Bessel beam generator (blue triangles).


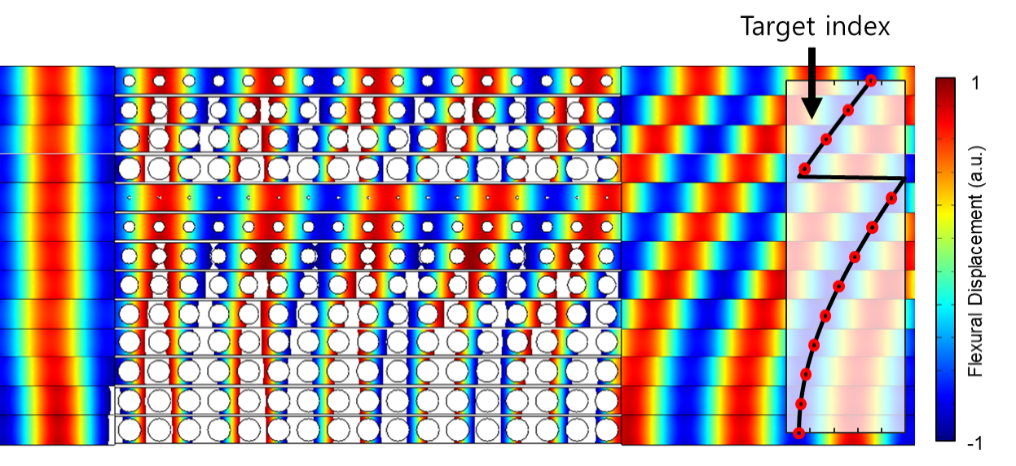


**Figure S3.** Layer-by-layer simulation results. Phase shifts in the layers with the radiuses calculated by the close form are simulated. Phases at the left-hand side are all in-phase and phases at the right-hand side shows the target phase we designed.

**S2. Designing Bessel beam generator**

Bessel beam generator is another example of the pGRIN-PC system. Bessel beam is one type of beams used in optical lens which has high aspect-ratio beam size. It can be created in theory by two plane waves directing opposite angles$\beta$and$-\beta$and thus the wave along the mirror axis does not spread out. The target refractive index of Bessel beam generator in this paper is defined with the refractive angle of $\beta={20}^{\circ}$ as

$n\left( y \right)=-\frac{1}{W}\left| y \right|\sin\left( \beta\right)+n(0)$ (S2)

Fig. S4(a) shows the refractive indices calculated for the layer locations. The corresponding radiuses for the layers can readily found by the closed form in Eq. (4) as in Fig. S2. Similar to the collimator case, each layer is simulated with the designed radiuses, and in Fig. S4(b), they successfully show the target phase difference to achieve ${20}^{\circ}$ refractive angle.


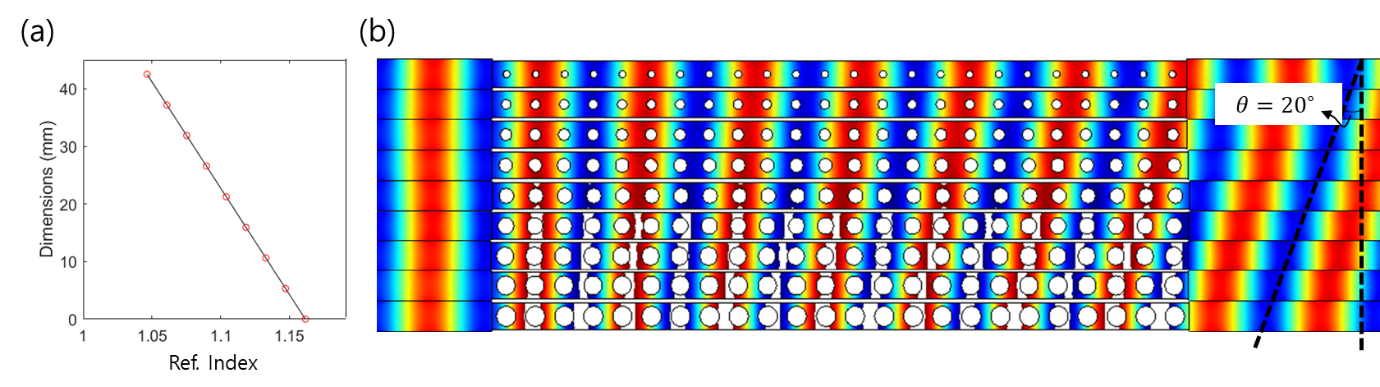


**Figure S4**. a) Refractive indices for the pGRIN-PC, and c) layer-by-layer simulation results.

**S3. Experiment setup and results**

The experiment setup is shown in Fig. S5. The pGRIN-PC consists of circular holes (scatterers) and line holes (partitions) which are manufactured by CNC (computerized numerical controlled) milling machine. The circular holes in 0.3 ~ 1.9 mm sizes were cut through, and then the 0.5 mm line holes are made. It is difficult to make the line holes in one cut, since 0.5 mm end-mill can be broken in the process. Thus, we first cut a groove of 0.7mm depth with the end mill and repeat the cut on the groove line until the line holes were made.

A source transducer of 50 kHz resonance frequency is attached to the back-side of the plate so that the vibrometer can scan the front-side. Laser Doppler Vibrometer (LDV) scans the flexural displacement normal to the plate in the front-side.

**
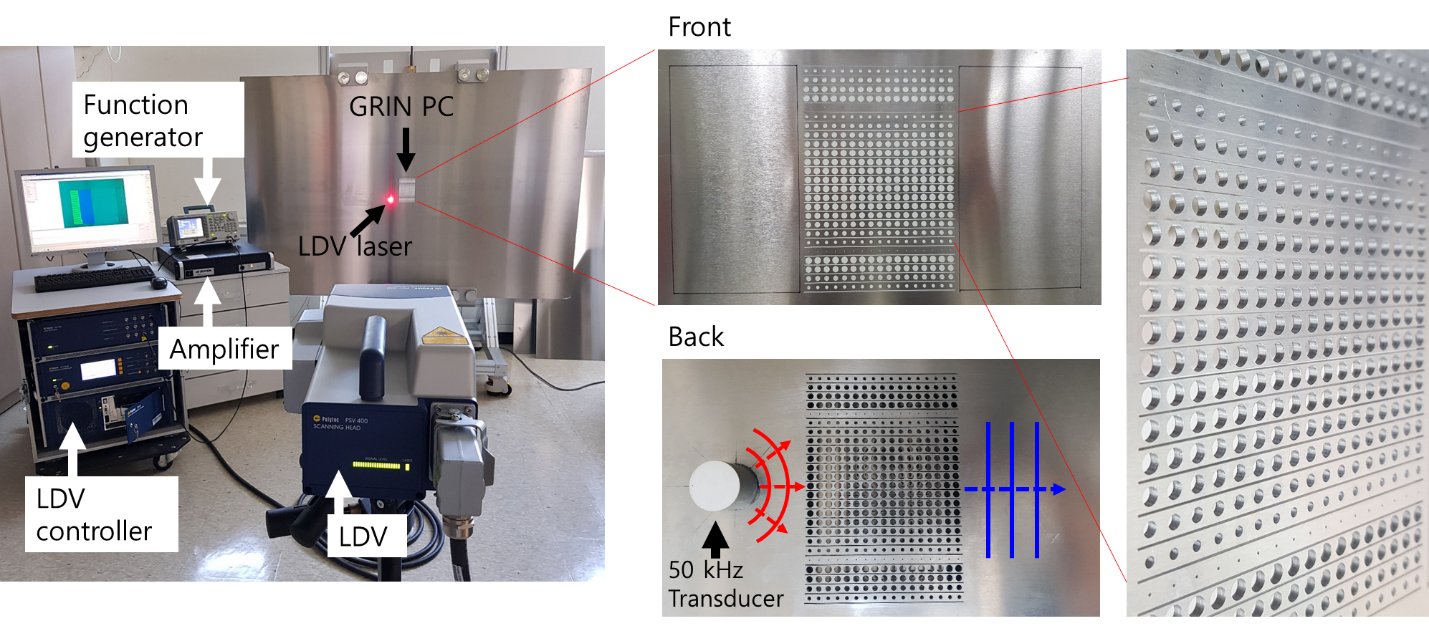
**

**Figure S5**. Experimental setup and the pGRIN-PC for the collimator. The source transducer is attached to the back-side of the plate so that the LDV can scan the area where the transducer is placed from the front-side.

For the collimator, since the pGRIN-PC has a fixed phase at each layer, reciprocity theorem must be guaranteed as mentioned in Section S1. Thus, we executed two different experiments. In the first experiment, input is a cylindrical source in the left-hand side, expecting a plane wave to be the transmitted in the right-hand side. In the second experiment, the input is a plane wave expecting focused wave as the output. The first experiment result is shown in Fig. S6 indicating that the waves are successfully collimated at the transmitted side from the cylindrical input wave and the second one in S7 showing that the waves are successfully focused at the focal point. This focusing effect is due to the fact that the graded lens acts as a phase compensator [2].

For the Bessel generator, incident plane wave is created towards the pGRIN-PC, and Fig. S8 show that the Bessel beam is generated from the plane wave input.


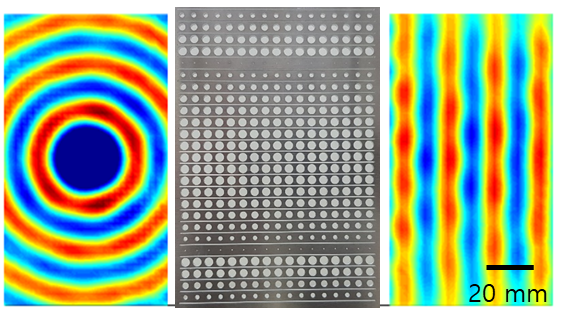


**Figure S6.** Snapshot of the experimental results for the collimator. The cylindrical source and the plane waves are clearly seen, respectively, at the left- and right-hand side of the collimator.


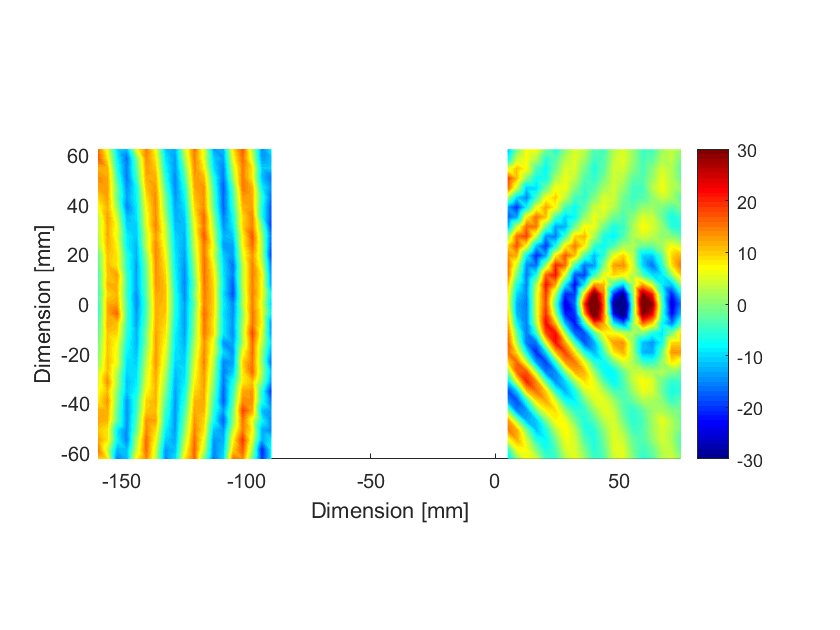

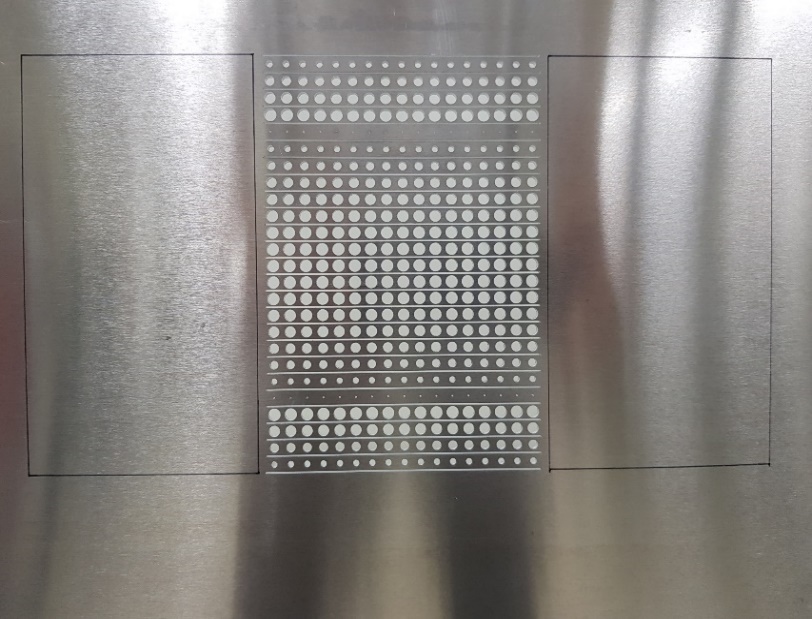


**Figure S7.** Snapshot of the experimental results for the focusing lens. The incident plate waves, and the waves focused at the focal point are clearly seen, respectively, at the left- and right-hand side of the collimator.


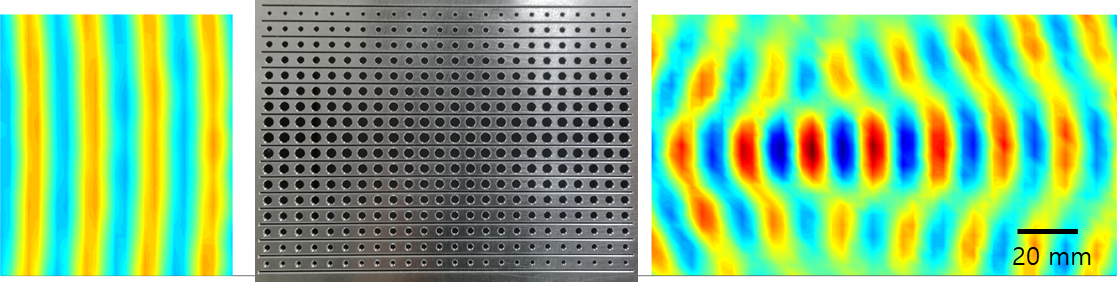


**Figure S8.** Snapshot of the experimental results for the Bessel beam generator**.** The plane wave source and the refracted waves are clearly seen, respectively, at the left- and right-hand side of the generator.

**Reference**

1. Wurmser, D. A., Manifestly Reciprocal Theory of Scattering in the Presence of Elastic Media. *Journal of Mathematical Physics*, **37**(9), 4434–4479 (1996)

2 Pinchuk, A. O. & Schatz, G. C. Metamaterials with Gradient Negative Index of Refraction. *Journal of the Optical Society of America A*, **24**(10) A39-A44 (2007)
